# Advanced Production, Processing and Characterization of Industrial Materials

**DOI:** 10.3390/ma18235366

**Published:** 2025-11-28

**Authors:** Jozef Mascenik, Tibor Krenicky

**Affiliations:** 1 Department of Computer Aided Manufacturing Technologies, Faculty of Manufacturing Technologies, Technical University of Kosice, Bayerova 1, 080 01 Presov, Slovakia; jozef.mascenik@tuke.sk; 2 Department of Manufacturing Process Control, Faculty of Manufacturing Technologies, Technical University of Kosice, Bayerova 1, 080 01 Presov, Slovakia

**Keywords:** production technologies, thermal processing, structure modelling, material surface, composites, material design, surface treatment, material quality test

## Abstract

This Special Issue presents recent advances in the production, modelling, processing, and characterization of advanced industrial materials, highlighting the diversity and sophistication of contemporary research discussing metallic, polymeric, composite, and nano-structured systems. The collected contributions address key challenges in materials science, ranging from surface quality control, the development of novel machining and fabrication tools, and optimization of thermoplastic composite consolidation, to provide fundamental insights into additive manufacturing, rheology, and constitutive modelling. The showcased studies introduce innovative approaches to metrology, including advanced optical, fluorescence, and X-ray scattering techniques for characterizing nano-particles, microstructures, and thermal properties. The presented research also features investigations into the welding of dissimilar steels, binder jetting of stainless steel, and the influence of heat treatment on functional steel performance, alongside environmentally oriented research on natural-fibre energy devices and bio-based polymer composites. Further research topics include defect structures in doped crystals, low-temperature synthesis of oxide films, and mechanical behaviour of steels under extreme conditions. Collectively, these articles demonstrate the strong synergy between experimental methods, computational modelling, and industrial applications, underscoring the continued progress in materials reliability, surface engineering, and advanced manufacturing technologies. This Special Issue therefore provides a comprehensive overview of current trends and emerging directions, offering valuable methodological and conceptual insights in the field.

## 1. Introduction

The rapid development of advanced industrial materials over the past two decades has significantly transformed modern manufacturing, structural engineering, and high-performance applications. Contemporary materials engineering is increasingly marked by the convergence of innovative production technologies, multi-scale computational modelling, and high-precision characterization tools, all aimed at enabling materials with tailored mechanical and functional properties [1,2,3,4]. The aerospace, automotive, biomedical engineering, and renewable energy sectors are increasingly relying on materials optimized for lightweight design, thermal stability, and reliability under complex service conditions [5,6].

Among recent technological advances, additive manufacturing (AM) represents a fundamental shift in production methodologies. Processes such as selective laser melting, electron beam melting, binder jetting, fused deposition modelling, and directed energy deposition allow near-net-shape fabrication of complex structures with controlled microstructural features [4,5,6,7]. Significant progress has been achieved in understanding melt pool dynamics, powder–laser interactions, solidification behaviour, and defect prevention, supported by advances in computational modelling and high-speed diagnostics [6,7,8,9]. Complementary research in AM process monitoring has introduced real-time optical, thermal, and acoustic approaches capable of detecting pores, spatter, and melt pool instabilities, laying the foundation for closed-loop control strategies [8,9,10,11,12].

Composite materials have likewise seen rapid development. Fibre-reinforced polymers, hybrid laminates, natural-fibre composites, and thermoplastic matrices are now widely used across modern engineering disciplines due to their high specific strength, corrosion resistance, and fatigue performance [13,14,15,16]. Thermoplastic composites in particular have benefited from new forming, consolidation, and automated placement technologies, supported by integrated thermal–mechanical modelling and improved control of crystallization and interlaminar bonding mechanisms [14,15,16,17].

Advances in surface engineering and high-resolution metrology have played a central role in enhancing material performance and reliability. Optical interferometry, confocal and laser profilometry, and interference microscopy provide increasingly detailed insight into topography, texture, and deformation at the micro- and nano-scale [12,18]. Progress in profilometric analysis has led to improved detection of wear, abrasion, and subsurface changes, particularly for advanced industrial alloys and tribological systems [19].

At smaller scales, nanomaterials research has been strengthened by powerful experimental tools for structural and chemical analysis, including small-angle X-ray scattering, advanced electron microscopy, and hybrid simulation approaches, which, together, enable precise characterization of nano-particle assemblies and defect structures [20,21]. In parallel, modern constitutive modelling frameworks—including viscoelastic, viscoplastic, and multi-physics formulations—support improved prediction of deformation, creep, and damage across a range of materials from polymers to metals [22,23].

Sustainability has grown into a central theme in materials research. Efforts to reduce environmental impact have accelerated the development of low-energy coating technologies, new corrosion-resistant systems, and environmentally resilient industrial materials [24,25]. At the same time, natural-fibre composites and bio-based modifiers have attracted increasing attention as renewable and recyclable alternatives for large-volume applications [16,26].

Artificial intelligence and data-driven approaches have become essential tools across manufacturing and materials science. AI-augmented modelling supports process–structure–property prediction, accelerates optimization, and enhances quality control, while enabling new forms of adaptivity in additive manufacturing and biomaterials development [27,28].

The current state of the art in advanced production, processing, and characterization of industrial materials is defined by stronger integration of modelling and experimentation, increased emphasis on in situ monitoring, the adoption of sustainable material systems, and the emergence of intelligent, data-driven design and manufacturing strategies. The significant engagement of the scientific community on this topic is evidenced by the numerous views on the articles, but more importantly by the rich citation response. To date, the articles in this Special Issue have been cited a total of 143 times, with an average of 8.4 citations per article, and this figure is continuing to rise, although the distribution of citations is considerably uneven, with review articles [29,30] standing out significantly so far.

## 2. Overview of Published Articles

This Special Issue presents a comprehensive view of current advances in industrial materials research. The articles included within collectively expand the knowledge on additive manufacturing, composite processing, advanced surface characterization, modelling, and sustainable materials engineering. Each study contributes to filling existing gaps—whether related to process optimization, joining of dissimilar materials, nano-scale metrology, or microstructural control. Simultaneously, this Special Issue outlines challenges that must be addressed to facilitate future research on advanced materials production, processing, and characterization.

### 2.1. Additive Manufacturing and Advanced Processing of Metallic Materials

In their study, Kráľ et al. (Contribution 8) applied binder jetting technology to 316L stainless steel, examining how different printing strategies affected both mechanical strength and dimensional accuracy. Their work emphasized the importance of post-processing and powder–binder interactions in determining final component performance. Similarly, Geľatko et al. (Contribution 9) investigated electron beam welding of dissimilar stainless and maraging steel joints, focusing on how welding parameters influenced microstructure, hardness distribution, and joint integrity. Their findings underscored the challenges of joining hybrid AM–conventional steel systems under industrial conditions. In a complementary theoretical contribution, Stejskal et al. (Contribution 17) developed a virial-theorem-based model to predict stiffness–density relationships in additively manufactured lattice structures. Their approach highlighted the potential of physics-based modelling to support design optimization in AM.

### 2.2. Composite Materials, Thermoplastic Processing, and Machining Technologies

Campos et al. (Contribution 3) analysed process parameters governing the continuous consolidation of thermoplastic composites in their statistical investigation, identifying optimal conditions for achieving consistent thickness and quality. Their results demonstrated how temperature, pressure, and speed interact in high-rate composite processing. Nomura et al. (Contribution 2) developed an electrodeposited wire-mesh grinding wheel for machining CFRP materials, emphasizing improved chip evacuation and reduced delamination during cutting. Their study highlighted the need for specialized tooling in advanced composite manufacturing. Acierno and Patti (Contribution 4) synthesize findings on material flow, thermal effects, and processing parameters to outline key challenges and opportunities in thermoplastic-based additive manufacturing using the principles and technological factors governing fused deposition modelling (FDM) of thermoplastic filaments, with particular emphasis on how rheological behaviour influences printability, process stability, and final part quality. Kulikov et al. (Contribution 15) explored the influence of epoxidized soybean oil on wood–polymer composites, demonstrating improvements in impact strength and dimensional stability, while emphasizing the potential of bio-based modifiers in sustainable composite development. In a related metallurgical context, Dudda (Contribution 16) evaluated the torsional behaviour of two steel grades at elevated temperatures, offering insights into thermomechanical stability under complex loading.

### 2.3. Surface Metrology, Materials Modelling, and Advanced Characterization

In his work on surface integrity, Ružbarský (Contribution 1) employed a laser profilometer to assess the surface roughness of AWJ-cut materials, highlighting the advantages of non-contact optical techniques for rapid, precise topography measurements. Complementing this, Staško et al. (Contribution 7) experimentally determined the emissivity of polished steel strips in continuous annealing lines, stressing the relevance of accurate thermal radiation data for non-contact temperature control in industrial heat treatment. Doveiko et al. (Contribution 6) advanced nano-particle metrology by combining dye fluorescence anisotropy, SAXS, and molecular dynamics to characterize silicate clusters, underscoring the need for multi-method approaches when dealing with nano-scale reactive systems. In a crystallographic study, Sidorov et al. (Contribution 12) examined homogeneous and gradient-doped LiNbO_3_:Er^3+^ crystals, revealing how defect structures manifest in IR transmission spectra and influence proton-related conductivity. At the modelling level, Guedes and Morais (Contribution 10) compared nonlinear constitutive models for epoxy resin behaviour, demonstrating that even minimally calibrated models can maintain predictive capability across creep, relaxation, and constant strain conditions. Nandipati et al. (Contribution 5), in their article, examine the convergence of nanomanufacturing and artificial intelligence, highlighting how data-driven and machine-learning methods enhance nano-scale fabrication, process optimization, defect prediction, and material behaviour modelling, framing AI as an enabling tool for next-generation nanomanufacturing systems and intelligent material processing workflows.

### 2.4. Environmentally Oriented Materials and Sustainable Processing

Barbaccia et al. (Contribution 13), in their contribution to sustainable surface engineering, describe a low-temperature method for forming copper oxide films under basic conditions, emphasizing cost- and energy-efficient processing routes. Varga et al. (Contribution 14) investigated a gravitational vortex turbine manufactured from natural fibres, analysing its hydrodynamic performance and identifying manufacturing-induced imbalances as key efficiency limitations. Their work demonstrated the challenges and opportunities associated with natural-fibre-reinforced components in small-scale renewable-energy technologies. The environmentally oriented composite study of Kulikov et al. (Contribution 15) (also referenced above) similarly highlighted the role of green additives in improving material performance without compromising sustainability.

### 2.5. Microstructure–Property Relations in Specialized Materials

In their microstructural analysis, Jia et al. (Contribution 11) studied SWP-B music steel wire subjected to various annealing treatments, revealing how cementite spheroidization and phase orientation influence both mechanical strength and acoustic performance. Their work demonstrated the close coupling of microstructure and functional properties in specialty steels. Additionally, the modelling study by Guedes and Morais (Contribution 10) provided valuable insight into the predictive reliability of time-dependent constitutive formulations for polymers, reinforcing the role of modelling in guiding material design.

## 3. Discussion and Conclusions

Despite the advancements described above, several important knowledge gaps remain that limit the full realization of advanced material technologies in industrial practice. Addressing these gaps is essential for improving reliability, sustainability, and design predictability in the field of industrial materials. For example, there is a need for more accurate multi-scale process–microstructure–property models. Although significant progress has been made, current models often fail to capture complex thermal–field interactions, phase transformations, porosity evolution, or fibre–matrix interfacial behaviour under dynamic processing conditions. For example, melt pool simulations in AM still struggle with capturing transient convection effects, while composite consolidation models inadequately predict interlaminar bonding at high processing speeds. Fully integrated multi-physics, multi-scale frameworks remain largely underdeveloped.

Another need concerns the implementation of real-time, standardized in-process monitoring. While advances in acoustic emission, thermography, pyrometry, digital image correlation, and AI-driven anomaly classification are encouraging, industrial adoption is limited. Many manufacturing processes still rely on post-manufacture inspection rather than continuous monitoring. This increases scrap rates, reduces manufacturing efficiency, and prevents the use of adaptive control strategies capable of correcting process deviations in real time.

In constitutive modelling, a major constraint is the limited availability of robust datasets needed for calibrating sophisticated models. Long-term behaviour, such as viscoelastic–viscoplastic coupling, environmental degradation, fatigue under variable loading, and moisture-induced changes, is still insufficiently understood, particularly for polymers and hybrid materials. The integration of machine learning helps alleviate data scarcity, but risks creating models with limited interpretability or restricted generalizability.

A persistent challenge also exists in the area of sustainable materials, where the variability of natural fibres, limited thermal stability, and incomplete understanding of degradation mechanisms hinder broader industrial deployment. Low-temperature or energy-efficient processes show promise but remain underexplored at scale. Lifecycle assessments for emerging materials are often incomplete, preventing informed decision-making regarding environmental trade-offs.

Surface integrity and defect characterization present another unresolved issue. As materials become increasingly complex—incorporating nano-modifiers, multi-phase microstructures, or functional gradients—traditional measurement techniques may lack the spatial resolution, environmental resistance, or quantitative reliability required to characterize subtle or multi-scale defects. High-temperature, in-line, or chemically reactive environments present additional challenges. A system-level gap also lies in the lack of comprehensive integrated design frameworks. Materials science, mechanical engineering, process optimization, sustainability analysis, and lifecycle evaluation often remain siloed disciplines. Without unified methodologies, materials may perform well in laboratory conditions but fail under real-world constraints related to manufacturability, durability, or environmental exposure.

Overall, while notable progress has been achieved, future work requires deeper interdisciplinarity and broader technological integration. Advances in modelling, sensing, the development of sustainable materials, and process standardization will be essential to address these gaps and to fully exploit the potential of next-generation industrial materials.
